# Single-cell transcriptomic and m6A methylation analyses reveal platelet-mediated immune regulatory mechanisms in sepsis

**DOI:** 10.3389/fimmu.2025.1607732

**Published:** 2025-06-23

**Authors:** Weiwei Qian, Yuanyuan Liu, Xinyang Zhao, Yuxin Dong, Jian Zhou, Songtao Shou

**Affiliations:** ^1^ Department of Emergency Medicine, Tianjin Medical University General Hospital, Tianjin, China; ^2^ Department of Immunology, International Cancer Center, Shenzhen University Health Science Center, Tianjin, China

**Keywords:** sepsis, single-cell transcriptomics, m6A methylation, platelets, RPA1, APP-CD74 signaling axis

## Abstract

**Objective:**

Sepsis is a systemic inflammatory response syndrome triggered by infection, characterized by high clinical heterogeneity and complex immunopathological mechanisms. Immune dysregulation plays a central role in its progression. This study aims to investigate the compositional changes of immune cells, characteristics of intercellular communication, and potential regulatory mechanisms of N⁶-methyladenosine (m^6^A) modification in sepsis, with a particular focus on the functional remodeling of platelets.

**Methods:**

This study integrated single-cell RNA sequencing data (GSE167363 dataset) from sepsis patients with m^6^A methylation sequencing data of peripheral blood mononuclear cells (PBMCs). Through systematic analysis, we compared the differences in immune cell composition, developmental trajectories, intercellular communication, and m^6^A modifications among healthy controls, survivors, and non-survivors, and further screened for key m^6^A-regulated target genes.

**Results:**

The analysis revealed that platelets gradually accumulated during the progression of sepsis, while B cells, T cells, and regulatory T cells (Tregs) exhibited a trend toward platelet-like phenotypic remodeling. Cell–cell communication analysis showed a marked decline in communication strength among immune cells as the disease worsened, particularly a significant weakening of the APP–CD74 signaling pathway between platelets and B cells, indicating impaired immune network synergy. m⁶A methylation sequencing revealed distinct remodeling of m⁶A peaks and dysregulation of related regulatory factors in non-survivors. Further integrative analysis identified RPA1 as a key m⁶A-regulated target gene, whose expression was closely associated with APP and co-regulated by multiple m^6^A-modifying factors.

**Conclusion:**

This study reveals disruptions in immune cell interactions and an m^6^A-dependent mechanism of platelet functional remodeling during sepsis progression. The identification of the key target gene RPA1 offers new insights into the immunopathological mechanisms of sepsis and lays a theoretical foundation for future precision interventions and therapeutic strategies.

## 1 Introduction

Sepsis is a life-threatening syndrome caused by a dysregulated host response to infection, often accompanied by multiple organ dysfunction (1–4). Despite advancements in antimicrobial therapy and organ support, sepsis continues to be associated with high morbidity and mortality, particularly among critically ill patients, where mortality rates can exceed 30% (5, 6). Increasing evidence suggests that the onset and progression of sepsis are closely linked to immune system dysregulation, with immune cell dysfunction considered one of the key determinants of clinical outcomes (4, 7).

Among the various immune cells, platelets have traditionally been recognized for their roles in hemostasis and thrombosis. However, recent studies have increasingly highlighted their emerging functions in immune regulation (8, 9). Platelets can actively participate in the immune response by releasing pro-inflammatory mediators, expressing immune molecules, and interacting with other immune cells (10). In the hyperinflammatory context of sepsis, platelets may not only contribute to microthrombus formation and tissue hypoperfusion but also promote inflammation and immune suppression through interactions with T cells and monocytes (11–15). Previous studies have demonstrated that sepsis can induce a spleen-derived population of protective platelets expressing high levels of CD40 ligand (1); platelet MHC class I molecules have been shown to impair CD8^+^ T cell function during sepsis (11); IFITM3 has been identified as a regulator of fibrinogen uptake and platelet reactivity in non-viral sepsis (16); and glycoprotein VI (GPVI) has been reported to enhance local immune defense in pneumococcal sepsis (17). These findings collectively underscore the crucial and multifaceted role of platelets in the immunoregulatory network of sepsis.

Epigenetic modifications, particularly N6-methyladenosine (m6A), the most prevalent and reversible internal modification of eukaryotic mRNA, have recently been recognized as critical regulators of immune responses and cell fate under various pathological conditions (18, 19). The m6A modification is dynamically regulated by “writers” (e.g., METTL3, METTL14), “erasers” (e.g., FTO, ALKBH5), and “readers” (e.g., YTHDF and IGF2BP families), which collectively influence mRNA stability, splicing, translation efficiency, and degradation (18, 19). Emerging evidence has revealed that m6A plays a pivotal immunoregulatory role in diverse diseases, including cancers, autoimmune disorders, and viral infections (20). In recent years, studies focusing on m6A in sepsis have gained momentum. For instance, histone protease-regulated METTL3 was shown to exacerbate sepsis-induced lung injury via m6A-mediated modification of ACSL4, promoting ferroptosis (21). Neutrophil extracellular traps (NETs) have been implicated in modulating m6A modification and regulating alveolar epithelial cell ferroptosis in sepsis-associated acute lung injury (22). METTL3 has also been reported to reprogram mitochondrial metabolism via an m6A–IGF2BP2–dependent mechanism, aggravating ferroptosis-induced myotoxicity (23). Additionally, YTHDF1 enhances WWP1 expression, promoting NLRP3 ubiquitination and thereby suppressing caspase-1–dependent pyroptosis (24). METTL3-mediated m6A modification also facilitates TLR4 pathway activation to enhance neutrophil activation during sepsis (25). Despite these advances in understanding the role of m6A in organ-specific damage during sepsis, its functional involvement in peripheral immune cells, particularly in the platelet-mediated immune regulatory network, remains poorly characterized.

Based on the aforementioned evidence, the present study integrates single-cell RNA sequencing (scRNA-seq) and m6A methylome sequencing to systematically investigate the immunoregulatory role of platelets in sepsis. We explore alterations at the levels of cellular composition, cell-cell communication, and epitranscriptomic modifications, aiming to identify key m6A regulatory factors and downstream target genes. This work provides novel mechanistic insights into the molecular underpinnings of sepsis and offers a theoretical foundation for the identification of potential therapeutic targets.

## 2 Materials and methods

### 2.1 Single-cell dataset and patient sample collection

This study utilized the publicly available scRNA-seq dataset GSE167363, which includes peripheral blood mononuclear cells (PBMCs) from 10 patients with Gram-negative sepsis (survivor group: n = 6; non-survivor group: n = 4) and 2 healthy controls (HCs) (26). Additionally, PBMCs from six sepsis patients (survivor group: n = 3; non-survivor group: n = 3), recruited from the Department of Emergency Medicine, Tianjin Medical University General Hospital, were used for m6A methylation sequencing. Informed consent was obtained from all participants, and the study protocol was approved by the Ethics Committee of Tianjin Medical University General Hospital (Approval ID: IRB2023-YX-045-01).

### 2.2 scRNA-seq data processing and quality control

Single-cell transcriptomic data were processed using the Seurat package (v5.0) in R. Low-quality cells were filtered out based on the following criteria: number of features < 200 or > 6000, and mitochondrial gene content > 15%. Data were normalized using the “LogNormalize” method, and the top 2000 highly variable genes were selected for downstream analysis. Principal component analysis (PCA) was performed for dimensionality reduction, with the number of components selected via elbow plot. Data integration across samples was conducted using Seurat’s canonical correlation analysis (CCA) to minimize batch effects. Louvain algorithm was employed for clustering, and visualization was performed using UMAP and t-SNE embeddings.

### 2.3 Cell type annotation and differential expression analysis

Cell type annotation was conducted using a combination of GPTCelltype (v1.1.1), SingleR (v1.0.0), and scCATCH (v3.1.1), with reference to established marker gene databases and published literature. Differentially expressed genes (DEGs) were identified using the wilcoxauc function from the RPresto package, with the significance threshold set at log_2_ fold change > 0.25 and adjusted p < 0.05. Functional enrichment analyses, including Gene Ontology (GO), Kyoto Encyclopedia of Genes and Genomes (KEGG), and Gene Set Enrichment Analysis (GSEA), were performed using clusterProfiler (v4.6.0).

### 2.4 Pseudotime trajectory analysis

To explore developmental dynamics, Monocle2 (v2.22.0) was applied to infer pseudotime trajectories of key immune cell subsets, including B cells, T cells, regulatory T cells (Tregs), and platelets, based on their transcriptional profiles.

### 2.5 Cell–cell communication analysis

Cell–cell communication networks were predicted using CellphoneDB (v2.1.7) to identify significant ligand–receptor interactions, with a minimum expression threshold of 10% set for inclusion in downstream analyses. NicheNet was used to assess ligand activity and predict target gene regulatory potential. The interaction network was visualized using the CCplotR R package.

### 2.6 m6A RNA methylation sequencing and experimental workflow

Total RNA was extracted from PBMCs using TRIzol™, followed by DNase I treatment and enzymatic purification. For m6A profiling, 20 μg of total RNA was fragmented in Mg^2+^ lysis buffer at 70°C for 6 minutes, and the reaction was terminated with EDTA. RNA fragments were purified using the Zymo RNA Clean & Concentrator-5 kit. m^6^A immunoprecipitation (MeRIP) was performed using an anti-m6A antibody and Protein A/G magnetic beads, incubated at 4°C for 2 hours. After elution and purification, sequencing libraries were constructed and subjected to paired-end sequencing (PE150) on the Illumina NovaSeq 6000 platform.

### 2.7 m6A methylation data analysis

Raw sequencing reads were processed using Cutadapt (v2.5) to trim adaptors and remove low-quality reads. Clean reads were aligned to the human genome (hg38) using Hisat2. Peak calling and differential methylation analysis were performed using exomePeak2 (v2.13.2), with a significance threshold of *p* < 0.05. Motif discovery and annotation were conducted using HOMER (v4.10.4). Differentially methylated genes were subjected to GO and KEGG enrichment analysis. Peak distribution and m^6^A site visualization were completed using Integrative Genomics Viewer (IGV, v2.19.1).

### 2.8 Candidate gene screening and correlation analysis

To identify key candidate genes, we integrated three datasets: (1) differentially m6A-modified genes in PBMCs; (2) DEGs in platelets between the survivor and non-survivor groups; and (3) DEGs in platelets between HCs and the survivor group. Genes shared across all three datasets were defined as core candidate genes. The correlation between the expression of these candidate genes and the platelet ligand APP was further assessed. In addition, we evaluated the correlation between candidate gene expression and 10 major m6A regulatory factors.

### 2.9 Statistical analysis and data visualization

All data visualizations were performed using R packages, including ggplot2, ComplexHeatmap, VennDiagram, and circlize. Statistical analyses were conducted based on data distribution and type, applying the Wilcoxon rank-sum test, Student’s t-test, or ANOVA as appropriate. A p-value < 0.05 was considered statistically significant.

## 3 Results

### 3.1 Single-cell transcriptomics reveals peripheral immune heterogeneity and a potentially critical role of platelets in sepsis progression

To investigate the immunological landscape of PBMCs in Gram-negative sepsis patients with distinct clinical outcomes (survivors *vs*. non-survivors), we performed scRNA-seq analysis based on the publicly available dataset GSE167363, which includes PBMCs from 10 sepsis patients (survivor group: n = 6; non-survivor group: n = 4) and 2 HCs.

**Figure 1:**
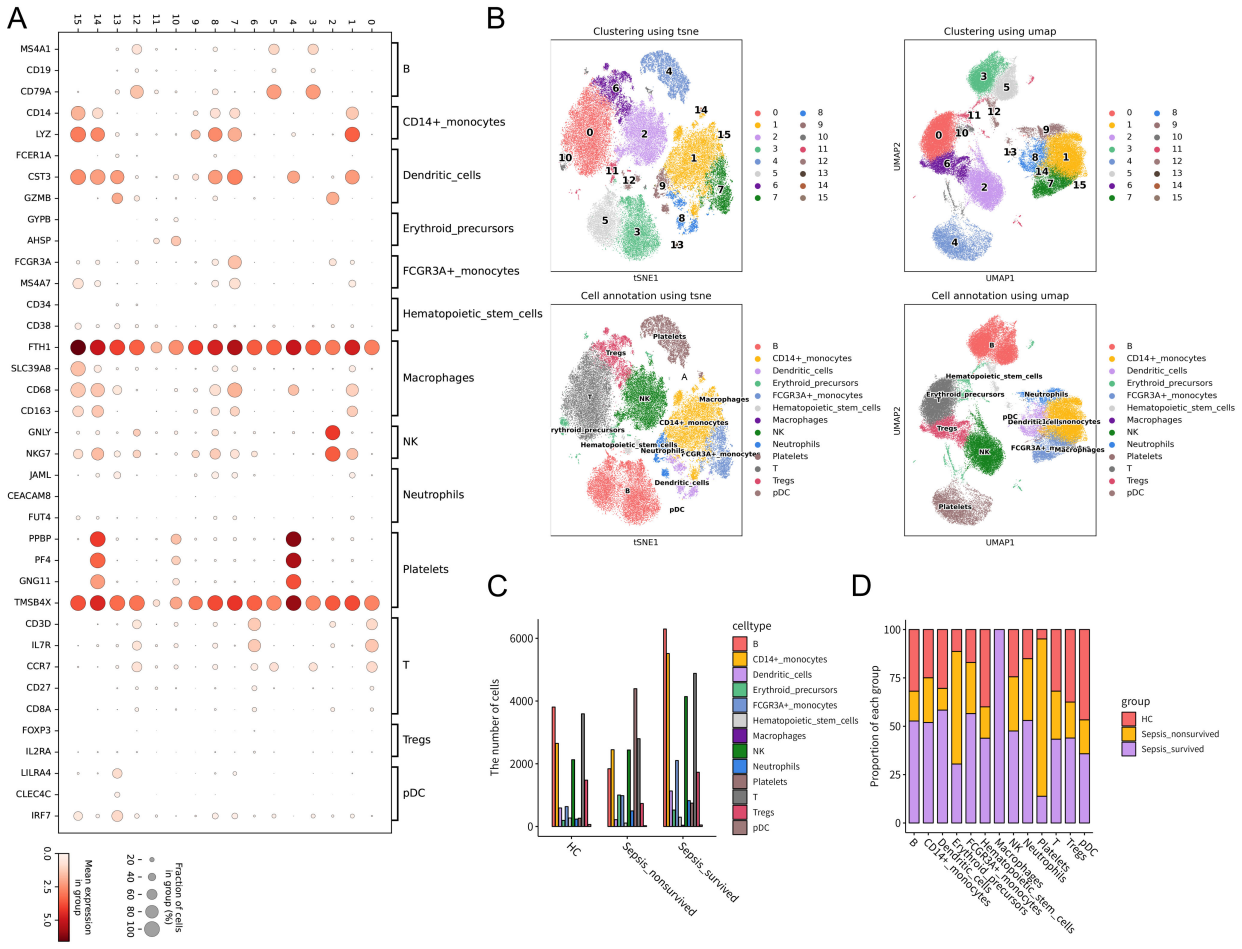
Single-cell transcriptomic analysis of peripheral blood mononuclear cells (PBMCs) from healthy controls (HCs) and patients with Gram-negative sepsis. Single-cell RNA sequencing was performed on PBMCs from 10 sepsis patients (survivor group: n = 6; non-survivor group: n = 4) and 2 healthy controls using the GSE167363 dataset. **(A)** Expression patterns of PBMCs-specific marker genes visualized in a bubble plot. Dot size represents the proportion of cells expressing a given marker, and color intensity indicates the average expression level. **(B)** Cell clustering was performed based on canonical marker expression, followed by dimensionality reduction and visualization using t-SNE and UMAP. **(C)** Comparison of total PBMCs counts across HCs, sepsis survivors, and non-survivors. **(D)** Relative proportions of immune cell subsets across the three groups, illustrating alterations in immune composition under different pathological conditions.

First, canonical immune cell marker genes were integrated to generate a bubble plot depicting marker gene expression patterns across different immune cell subsets (**Figure 1A**). Based on these expression profiles, unsupervised clustering was performed, followed by dimensionality reduction using t-SNE1 and UMAP1, leading to the successful identification of multiple PBMCs subpopulations, including B cells, CD14+ monocytes, FCGR3A+ monocytes, dendritic cells (DCs), T cells, regulatory T cells (Tregs), natural killer (NK) cells, platelets, erythroid progenitors, neutrophils, macrophages, plasmacytoid dendritic cells (pDCs), and hematopoietic stem cells (**Figure 1B**). Notably, the distribution of these cell subsets varied significantly among HCs, survivors, and non-survivors, indicating pronounced immune heterogeneity across groups (**Supplementary Figures 1A–D**).

At the cellular level, a comparison of total PBMCs counts among the HC, survivor, and non-survivor groups revealed distinct patterns of cellular abundance (**Figure 1C**, **Supplementary Table S1**). Further analysis of immune cell composition showed a progressive increase in the relative proportion of platelets from HCs to survivors and non-survivors (**Figure 1D**). This trend suggests that platelets may not only participate in the immune response during sepsis but also be closely associated with disease severity and clinical outcomes. Taken together, these findings indicate that the immune cell composition of PBMCs in Gram-negative sepsis patients undergoes significant alterations. In particular, the increasing proportion of platelets during disease progression suggests their potential key role in the pathogenesis and adverse outcomes of sepsis.

### 3.2 Dynamic changes in platelet differentiation trajectories and immune communication networks in sepsis

**Figure 2:**
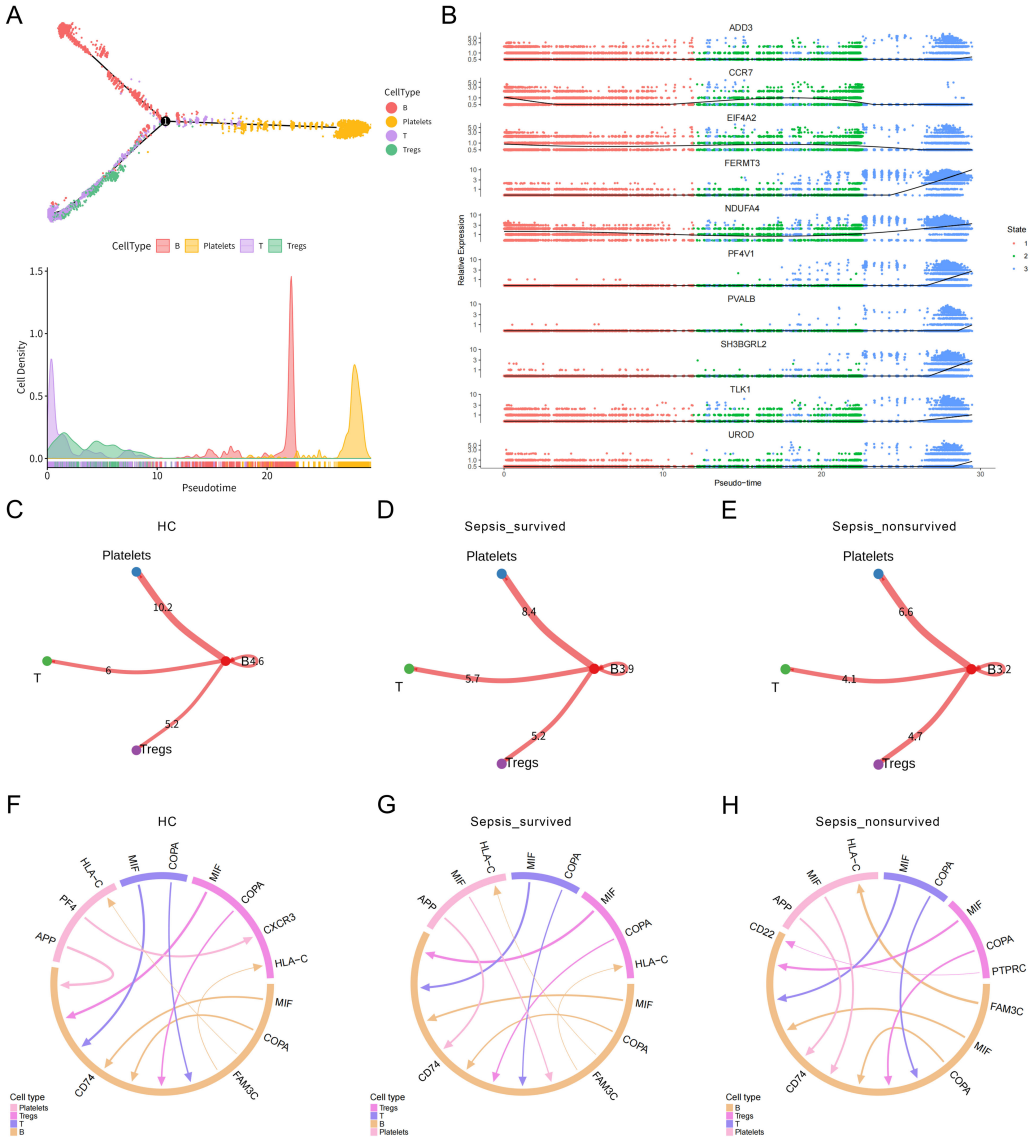
Pseudotime trajectory and cell–cell communication analysis of immune cell differentiation toward platelets. **(A)** Pseudotime trajectory analysis of peripheral blood mononuclear cells (PBMCs) illustrating immune cell developmental dynamics. Cells in different states are color-coded, revealing a potential differentiation trajectory from B cells, T cells, and Tregs toward a platelet-like phenotype. **(B)** Dynamic changes in gene expression profiles during the transition of B cells, T cells, and Tregs toward platelet-like states. **(C–E)** Heatmaps of overall cell–cell interaction strength among immune cell subsets in **(C)** healthy controls (HCs), **(D)** sepsis survivors, and **(E)** sepsis non-survivors. **(F–H)** Ligand–receptor interaction networks between B cells, Tregs, T cells, and platelets in the three groups.

To further investigate the immunological potential of platelets in sepsis, we performed pseudotime trajectory analysis on PBMCs. The results revealed that B cells, T cells, and Tregs exhibited continuous and well-defined developmental trajectories along the pseudotime axis, ultimately converging toward a platelet-like phenotype (**Figure 2A**, **Supplementary Figures 2A–C**). These findings suggest that, under septic conditions, certain immune cells may undergo phenotypic reprogramming or functional transdifferentiation, acquiring platelet-like features. This differentiation process was accompanied by dynamic changes in gene expression profiles within these cell subsets, further supporting the presence of a platelet-directed reprogramming trajectory (**Figure 2B**).

To assess potential intercellular communication between immune cells and platelets, we constructed and analyzed the PBMCs cell–cell interaction network. Global interaction strength analysis indicated a progressive weakening of immune cell communication with advancing disease severity, with the non-survivor group exhibiting the sparsest interaction network (**Figures 2C–E**). Focusing on the ligand–receptor interactions between B cells, T cells, Tregs, and platelets, we observed that in healthy individuals, communication between these subsets and platelets was relatively active (**Figure 2F**). However, in the sepsis survivor group (**Figure 2G**) and the non-survivor group (**Figure 2H**), the network became increasingly impaired. Notably, the signaling intensity of the APP → CD74 axis, a key communication route between platelets and B cells, decreased progressively across groups: 10.88 in the healthy control group, 8.864 in the survivor group, and 6.636 in the non-survivor group. This trend indicates that platelet–B cell signaling is markedly suppressed during sepsis progression. Given the critical role of CD74 as both a chaperone of MHC class II molecules and a regulator of inflammatory signaling, attenuation of this axis may reflect impaired immune activation or defects in antigen presentation capacity. Taken together, these findings reveal that the immune communication network between platelets and immune cells undergoes substantial remodeling during the course of sepsis. The pronounced weakening of this network, particularly in non-survivors, suggests a functional impairment of platelets in maintaining immune homeostasis, which may contribute to immune dysregulation and poor clinical outcomes in sepsis.

### 3.3 Differential gene expression analysis reveals functional enrichment of platelets in sepsis

**Figure 3:**
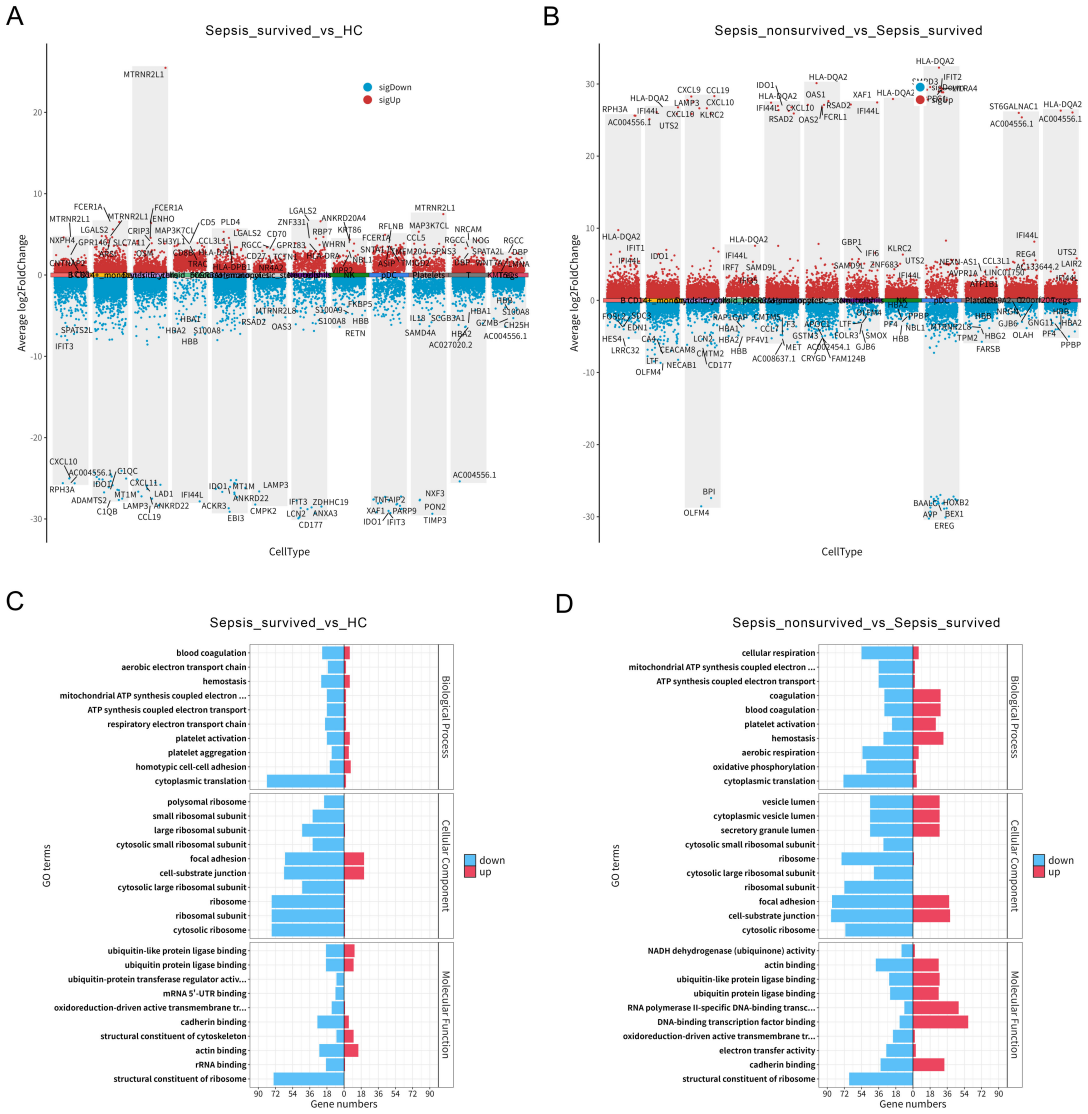
Differential gene expression and platelet functional enrichment in peripheral blood mononuclear cells (PBMCs) of sepsis patients. **(A)** Differential gene expression analysis across PBMCs subsets between healthy controls (HCs) and sepsis survivors. **(B)** Differential gene expression across PBMCs subsets between sepsis survivors and non-survivors. **(C)** Gene Ontology (GO) enrichment analysis of differentially expressed genes (DEGs) in platelets between HCs and sepsis survivors. **(D)** GO enrichment analysis of platelet DEGs between sepsis survivors and non-survivors.

To further explore the functional alterations of PBMCs under different clinical outcomes in sepsis, we first performed differential gene expression analysis across immune cell subsets between HCs and the sepsis survivor group (**Figure 3A**, **Supplementary Table S2**). The results revealed extensive transcriptomic differences across multiple immune cell populations, suggesting that sepsis induces widespread immune activation and functional reprogramming in specific cell subsets. Subsequent comparison of PBMCs between the survivor and non-survivor groups revealed more pronounced transcriptional alterations in the non-survivors across several immune cell types (**Figure 3B**, **Supplementary Table S3**), particularly in B cells, T cells, and platelets. These findings indicate that these key immune populations may undergo functional transitions that are closely associated with disease progression.

To better understand the functional changes in platelets during sepsis, we conducted GO enrichment analysis of DEGs between HCs and survivors (**Figure 3C**), as well as between survivors and non-survivors (**Figure 3D**). The results showed that platelet DEGs in sepsis were significantly enriched in biological processes such as blood coagulation, platelet activation, and hemostasis, indicating an enhanced activation of their classical hemostatic functions. Notably, these functional enrichments were further intensified in the non-survivor group, suggesting that in addition to their conventional roles, platelets may be actively involved in inflammatory responses and immune dysregulation in advanced stages of sepsis. In summary, our data reveal substantial transcriptomic reprogramming of monocytes and platelets within the PBMCs compartment of sepsis patients. Specifically, platelets exhibit a progressively enhanced functional state across groups with worsening clinical outcomes, highlighting their potential key role in the immunopathogenesis of sepsis.

### 3.4 m6A methylation profiles and functional enrichment in PBMCs of sepsis patients

**Figure 4:**
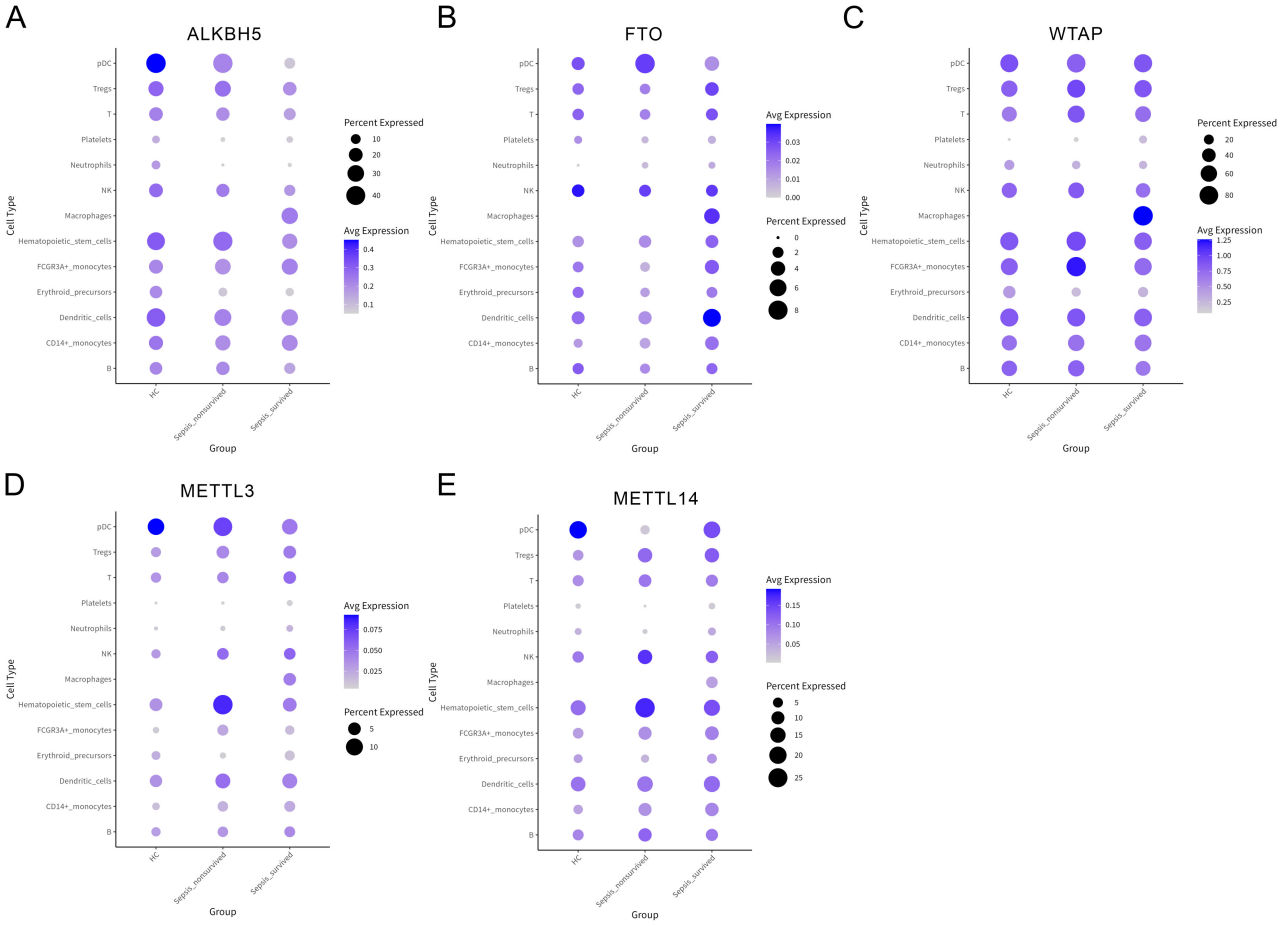
Expression profiles of N6-methyladenosine (m6A) regulatory enzymes across peripheral blood mononuclear cells (PBMCs) subsets. **(A–E)** Bubble plots showing the expression levels of key m6A regulatory factors in various PBMCs subsets, including ALKBH5 **(A)**, FTO **(B)**, WTAP **(C)**, METTL3 **(D)**, and METTL14 **(E)**.

Previous analyses revealed a progressive weakening of immune communication between platelets and B cells during sepsis; however, the underlying molecular mechanisms remain unclear. To investigate the role of m6A in this process, we first assessed the expression patterns of canonical m6A regulatory enzymes across different PBMCs subsets in HCs, sepsis survivors, and non-survivors (**Figures 4A–E**, **Supplementary Figures 3A–C**). We observed substantial heterogeneity in the expression of demethylases (ALKBH5, FTO) and methyltransferase complex components (WTAP, METTL3, METTL14), suggesting their potential involvement in the epigenetic regulation of immune function during sepsis.

**Figure 5:**
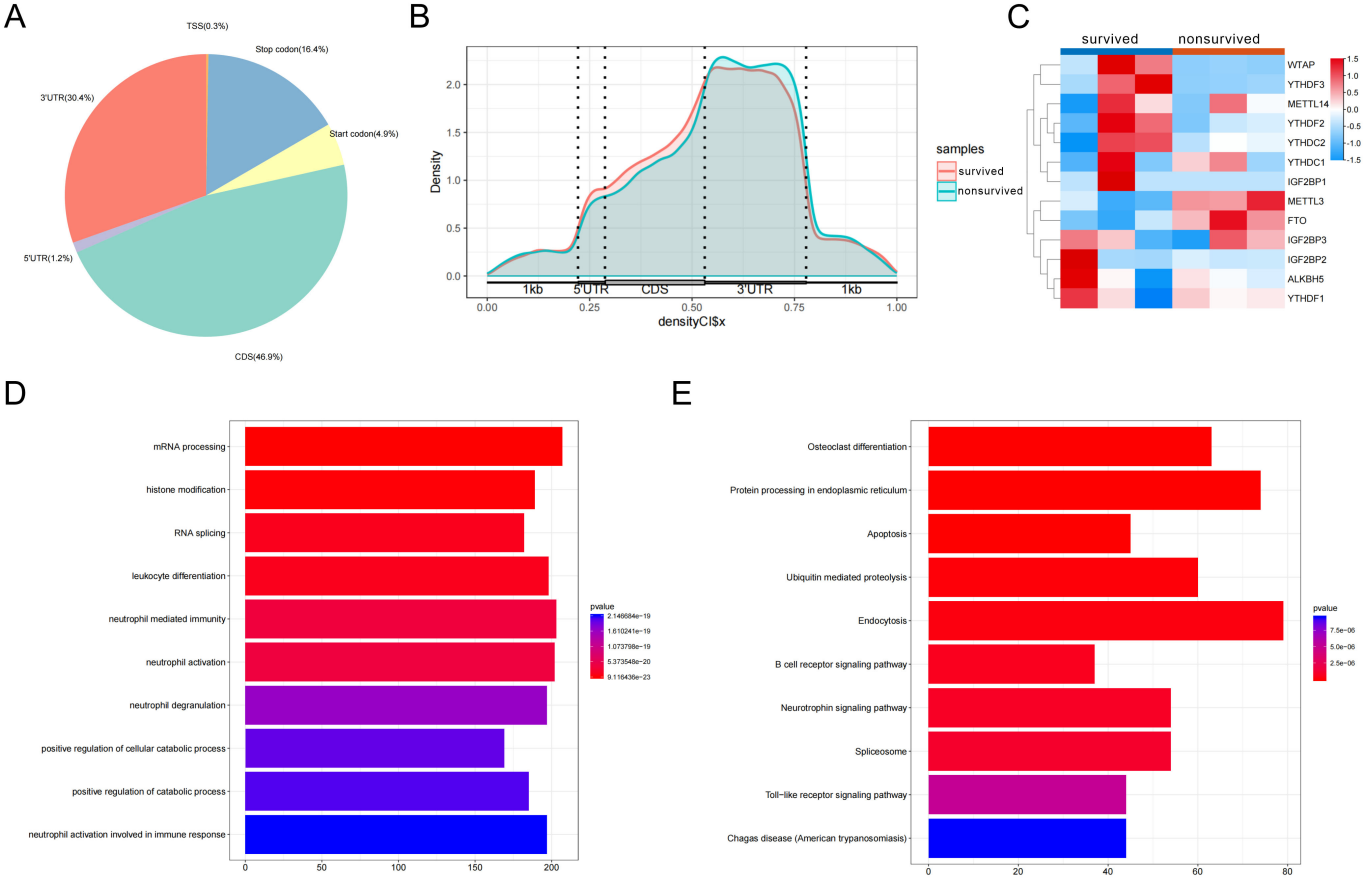
N6-methyladenosine (m6A) methylation landscape and functional enrichment analysis in peripheral blood mononuclear cells (PBMCs) from sepsis survivors and non-survivors. **(A)** Pie chart depicting the overall distribution of m6A peaks across mRNA transcripts in PBMCs from sepsis survivors (n = 3) and non-survivors (n = 3), based on MeRIP-seq analysis. **(B)** Average distribution of m6A peaks across transcript regions, including 5’UTR, coding sequence, and 3’UTR, in the survivor and non-survivor groups. **(C)** Heatmap illustrating differential expression of m6A regulatory enzymes in PBMCs between sepsis survivors and non-survivors. **(D, E)** Functional enrichment of differentially methylated genes based on Gene Ontology annotation **(D)** and Kyoto Encyclopedia of Genes and Genomes pathway analysis **(E)**.

Next, we performed MeRIP-seq on PBMCs from survivors and non-survivors. In both groups, m6A peaks were predominantly enriched in coding sequence (CDS) regions (46.9%), followed by 3′ untranslated regions (3′UTR) (30.4%) (**Figure 5A**). Notably, a slight reduction in CDS-localized m6A peaks was observed in the non-survivor group compared to survivors (**Figure 5B**), implying that disease progression may alter m6A distribution and affect mRNA stability or translational efficiency. Differential expression analysis revealed that several m6A regulators were significantly dysregulated in the non-survivor group (**Figure 5C**), further supporting the dynamic involvement of m6A modification in sepsis pathogenesis. Functional enrichment analysis of differentially methylated genes revealed significant associations with pathways related to metabolic regulation, inflammatory responses, apoptosis, and immune signaling (**Figures 5D, E**), indicating that m6A modification may influence sepsis through multilayered regulatory mechanisms. In summary, sepsis patients exhibit dynamic remodeling of m6A methylation in PBMCs, characterized by altered regulator expression, redistribution of modification sites, and functional pathway enrichment. These results highlight the potential contribution of m6A-dependent regulation to disease progression and outcome in sepsis.

### 3.5 1.10 m6A-regulated characteristics of RPA1 and its correlation with key immune factors in sepsis

**Figure 6:**
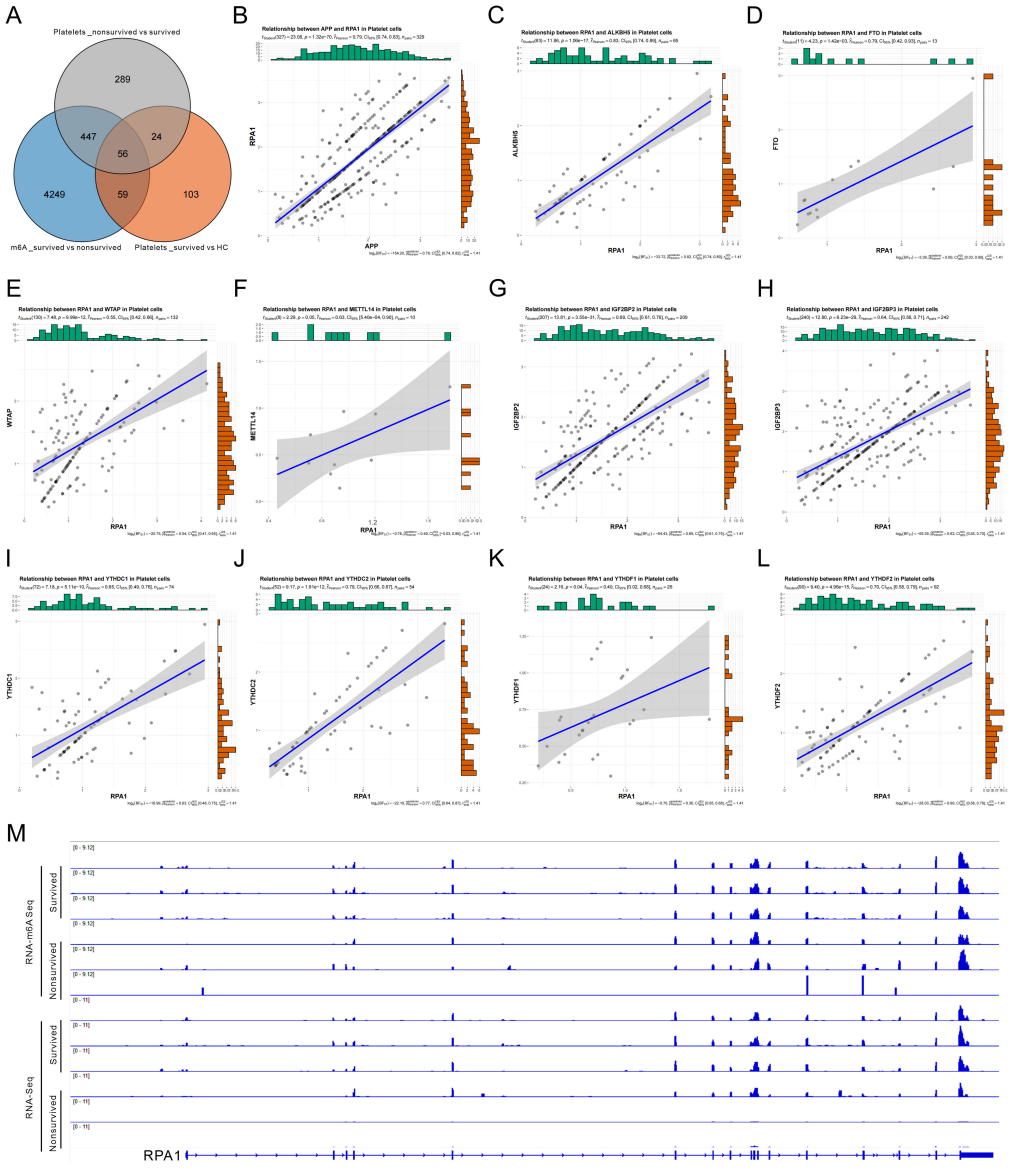
N6-methyladenosine (m6A)-mediated regulation of RPA1 and its correlation with key factors in sepsis. **(A)** Venn diagram showing the intersection of three sets of differentially regulated genes: (1) differentially m6A-methylated genes in peripheral blood mononuclear cells (PBMCs) between sepsis survivors and non-survivors; (2) differentially expressed genes (DEGs) in platelets between survivors and non-survivors; and (3) DEGs in platelets between healthy controls (HC) and survivors. **(B)** Correlation analysis between RPA1 and the platelet-specific ligand APP. **(C–L)** Correlation analysis between RPA1 and ten key m6A regulatory factors in platelets, including ALKBH5 **(C)**, FTO **(D)**, WTAP **(E)**, METTL14 **(F)**, IGF2BP2 **(G)**, IGF2BP3 **(H)**, YTHDC1 **(I)**, YTHDC2 **(J)**, YTHDF1 **(K)**, and YTHDF2 **(L)**. **(M)** Integrative Genomics Viewer tracks showing differential m6A methylation abundance and RPA1 expression levels in PBMCs from sepsis survivors versus non-survivors.

To identify key genes potentially involved in m6A-mediated immune remodeling in sepsis, we integrated three sets of differentially expressed or modified genes for intersection analysis: (1) Differentially methylated genes between sepsis survivors and non-survivors in PBMCs; (2) DEGs in platelets between survivors and non-survivors; (3) Differentially expressed platelet genes between HCs and survivors. This integrative analysis yielded 56 overlapping candidate genes (**Figure 6A**). To evaluate their potential role in platelet–immune cell interaction, particularly in relation to B cell communication, we examined the correlation between candidate genes and the platelet-derived ligand APP. RPA1 exhibited the strongest correlation with APP expression (**Figure 6B**, **Supplementary Table S4**), suggesting a possible role in the APP–CD74 signaling axis and platelet–B cell interaction.

Further correlation analysis revealed that RPA1 expression in platelets was significantly associated with multiple m6A regulators, including demethylases (ALKBH5, FTO), methyltransferase complex components (WTAP, METTL14), and reader proteins (IGF2BP2/3, YTHDC1/2, YTHDF1/2) (**Figures 6C–L**), indicating that RPA1 may be epigenetically regulated by m6A modification. Consistently, IGV browser visualization demonstrated reduced m6A modification levels and decreased RPA1 expression in PBMCs from the non-survivor group compared to survivors (**Figure 6M**), further supporting RPA1 as a putative m6A-regulated target gene in sepsis. Taken together, RPA1 emerged as a key candidate gene identified through cross-dataset integration. It is closely correlated with the platelet ligand APP and subject to regulation by multiple m6A factors. These findings suggest that RPA1 may contribute to the regulation of immune cell communication in sepsis and holds promise as a mechanistic mediator and potential therapeutic target.

## 4 Discussion

In this study, we systematically characterized the immunological landscape, platelet functional remodeling, and epigenetic regulatory mechanisms in PBMCs from sepsis patients with different prognostic outcomes using scRNA-seq and MeRIP-seq profiling. Our findings highlight the crucial role of platelets and m6A modification in the pathogenesis and progression of sepsis. Traditionally recognized for their roles in hemostasis and thrombosis, platelets are increasingly acknowledged as active immune regulators in sepsis (2, 27, 28). Previous studies have demonstrated that platelets can participate in immune defense by expressing immunomodulatory molecules such as P-selectin and CD40 ligand (CD40L), and by releasing inflammatory mediators, thereby contributing to pathogen recognition, immune cell recruitment, and amplification of inflammatory signaling (29–33). In our study, we observed a significant increase in the proportion of platelets as sepsis progressed, accompanied by a marked decline in platelet–B cell communication in the non-survivor group. These findings further emphasize the potential involvement of platelets in immune dysregulation and poor clinical outcomes during sepsis.

In recent years, m6A methylation, the most prevalent internal modification of eukaryotic mRNA, has been increasingly implicated in the regulation of inflammation and immune-related disorders (34–37). Although studies on m6A modification in the context of sepsis remain limited, emerging evidence suggests that m6A modulates cytokine expression, immune cell activation, and inflammatory responses (38–40). In this study, MeRIP-seq analysis revealed a reprogramming of the m6A epitranscriptome in PBMCs from non-survivors, with enhanced m6A enrichment in CDS regions, suggesting potential impacts on mRNA stability and translational efficiency. Moreover, we identified significant differential expression of several m6A regulators among different prognostic groups, including methyltransferases (METTL3, METTL14), demethylases (FTO, ALKBH5), and reader proteins such as YTHDF1 and IGF2BP2. These findings indicate a dynamic regulatory landscape of m6A modification in sepsis and highlight the potential of m6A-related enzymes as therapeutic targets for modulating immune responses in sepsis.

Of particular interest is the APP → CD74 signaling axis, which exhibited a progressively decreasing trend in our cell–cell communication analysis and has also been implicated in various immune-related diseases (41). For example, APP–CD74 interactions have been shown to mediate endothelial–macrophage communication, thereby promoting renal injury and fibrosis (41). Therapeutic modulation of this axis has been reported to enhance the phagocytic activity of tumor-associated macrophages (TAMs) in glioblastoma (GBM) (42). Single-cell transcriptomic analysis has further revealed the involvement of the APP–CD74 axis in reinforcing immune suppression and tumor progression in testicular cancer (43). Additionally, APP-CD74 interactions have been shown to inhibit β-amyloid production, suggesting a potential multifaceted role for this pathway in inflammatory and metabolic environments (44). However, investigations into the function of the APP → CD74 axis in sepsis remain limited. To our knowledge, this study is the first to report a significant attenuation of this signaling axis at the single-cell level in sepsis, correlating with poor clinical outcomes. This novel insight warrants further exploration.

Moreover, RPA1, identified as a candidate gene at the intersection of differential m6A modification and platelet-specific expression, may play a key role in platelet immune function and the epigenetic regulation of sepsis. Recent evidence has shown that RPA1, the core subunit of the single-stranded DNA-binding protein complex, plays a critical role in fundamental cellular processes, including DNA replication, damage repair, chromatin accessibility, and telomere maintenance (45). Loss of RPA1 not only compromises genomic stability but also promotes necroptotic death of T cells by activating the ZBP1-RIPK3 signaling pathway, leading to immune dysregulation and exacerbated inflammatory responses. These findings have been validated in the contexts of immune homeostasis, inflammatory diseases, and tumor immune tolerance (46–50). In addition, aberrant expression of RPA1 has been implicated in various pathological conditions, including ulcerative colitis, lipid metabolism disorders, radioresistant cancers, and neurodegenerative diseases. Its multifaceted regulatory functions have been widely reported across multiple biological systems (51, 52). However, the specific role of RPA1 within the platelet-immune regulatory network in sepsis remains largely unexplored and lacks direct experimental evidence. Our study found that RPA1 expression was strongly correlated with the platelet-derived ligand APP, and showed co-expression with several m6A regulators, including METTL3 and ALKBH5, suggesting that its expression and function may be subject to dynamic m6A-dependent modulation. Given the observed attenuation of the APP → CD74 axis in the non-survivor group, we hypothesize that RPA1 may contribute to the regulation of this ligand–receptor pair, thereby influencing immune cell activation and inflammatory signaling. This, in turn, could mediate functional reprogramming of platelets during sepsis. It is important to note that although this study is the first to propose RPA1 as a potential molecular link between platelet immune function and m^6^A modification, the regulatory mechanisms inferred herein are primarily based on bioinformatic correlation analyses. Direct molecular and functional validation is currently lacking. We fully acknowledge the need for future investigations employing functional experiments in cellular and animal models—such as RPA1 knockdown or overexpression—to elucidate its specific effects on platelet-immune cell interactions, cytokine secretion, and immune homeostasis. Additionally, experimental validation of the APP→CD74 signaling axis is necessary to determine whether RPA1 is involved in modulating this key ligand-receptor pathway. Furthermore, the integration of advanced technologies such as CRISPR-based gene editing, single-cell multi-omics, and high-throughput epigenomic profiling may offer deeper mechanistic insights into the role of RPA1 in platelet-mediated immune regulation and sepsis progression.

Despite uncovering a range of critical cellular dynamics, intercellular communication patterns, and epigenetic regulatory features, this study has several limitations. First, the relatively small sample size, with some analyses based on a limited number of patient samples, may reduce statistical power and limit the generalizability of the findings. Second, m6A methylation data were derived from bulk PBMCs populations, which restricts the resolution of m6A modification dynamics at the single-cell level. Third, the mechanistic inferences drawn from this study are primarily based on computational predictions, and lack direct experimental validation. Future investigations should aim to validate these findings in larger, multi-center cohorts, and leverage emerging technologies such as CRISPR-based epigenetic editing, CLIP-seq, and single-cell m6A sequencing techniques (e.g., DART-seq) to dissect the cell type-specific functions of m6A in platelets with higher precision. Additionally, the APP → CD74 signaling axis and the RPA1-centered regulatory network warrant in-depth experimental exploration to evaluate their potential as targets for therapeutic intervention in sepsis.

In summary, this study reveals significant immune cell heterogeneity, platelet immune reprogramming, and m6A-mediated gene regulation in the peripheral blood of sepsis patients. Notably, we identify RPA1 as a potential key node linking platelet immune function with m6A epitranscriptomic remodeling. These findings offer new epigenetic insights into the pathogenesis of sepsis, and lay the groundwork for future research aimed at biomarker discovery and targeted therapeutic strategies.

## Data Availability

The datasets presented in this study can be found in online repositories. The names of the repository/repositories and accession number(s) can be found in the article/**Supplementary Material**.
