# Supplementary figures and images for: Single-cell transcriptomic and m6A methylation analyses reveal platelet-mediated immune regulatory mechanisms in sepsis
Source: Front Immunol. 2025 Jun 23;16:1607732. doi: 10.3389/fimmu.2025.1607732 (PMC12230005; doi:10.3389/fimmu.2025.1607732)

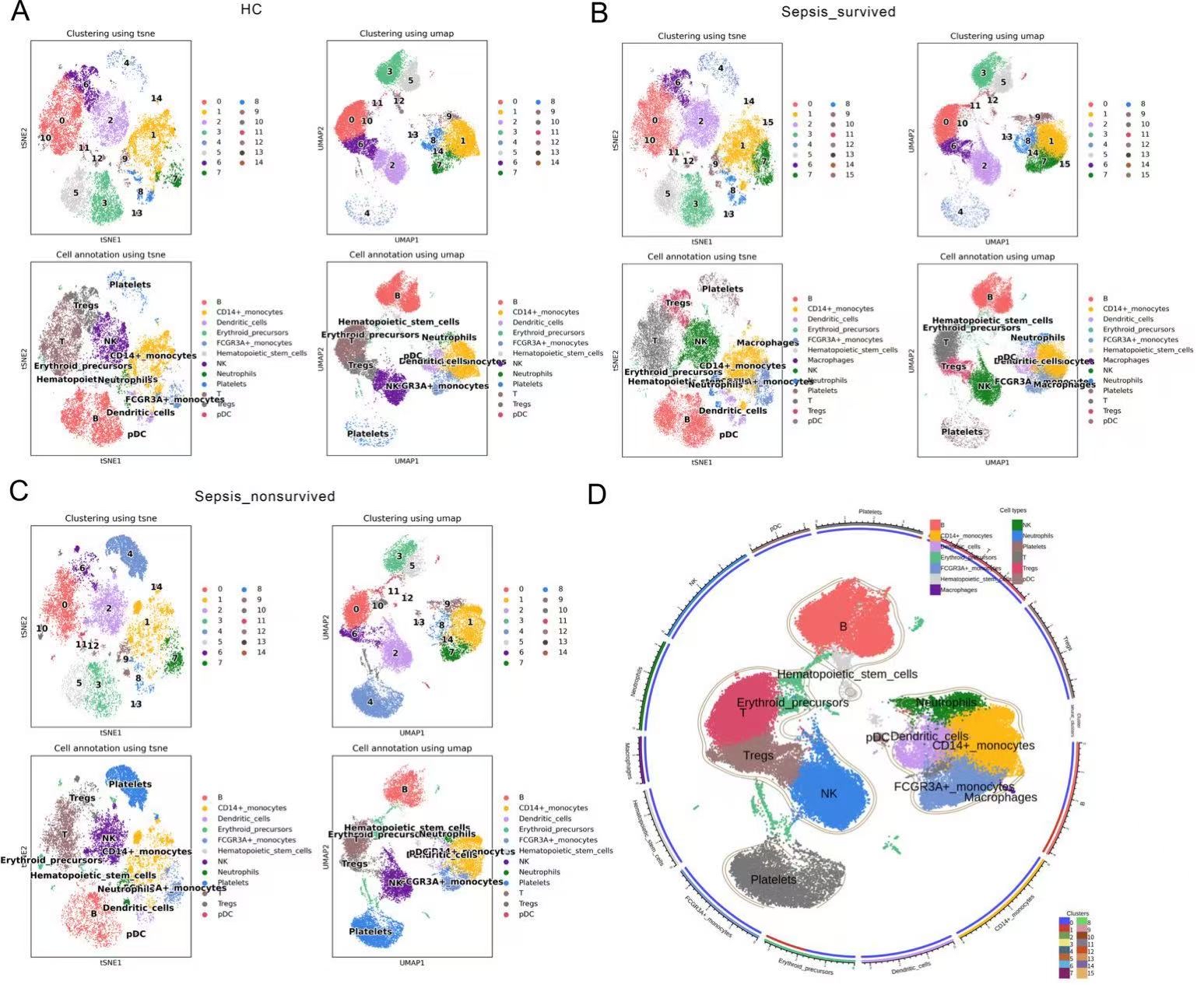

Supplement: Supplementary file 1 [file Image1.jpeg]

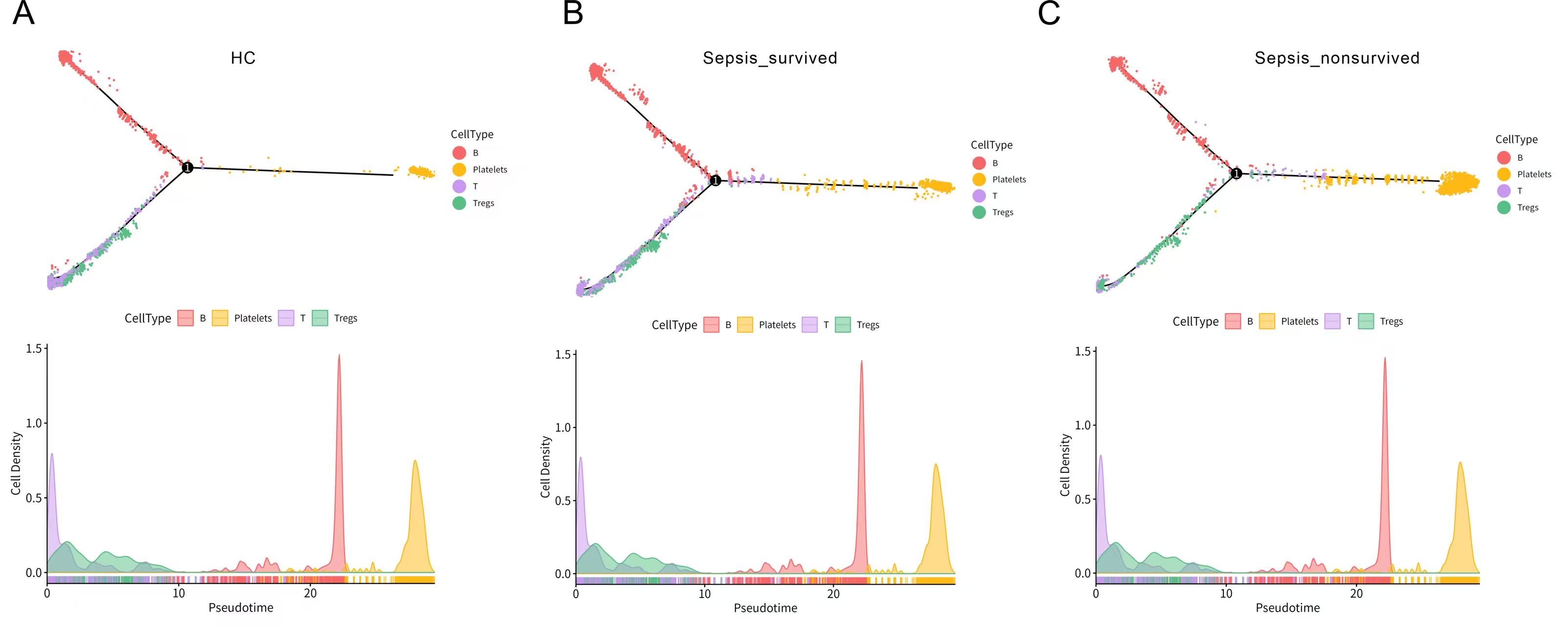

Supplement: Supplementary file 2 [file Image2.jpeg]

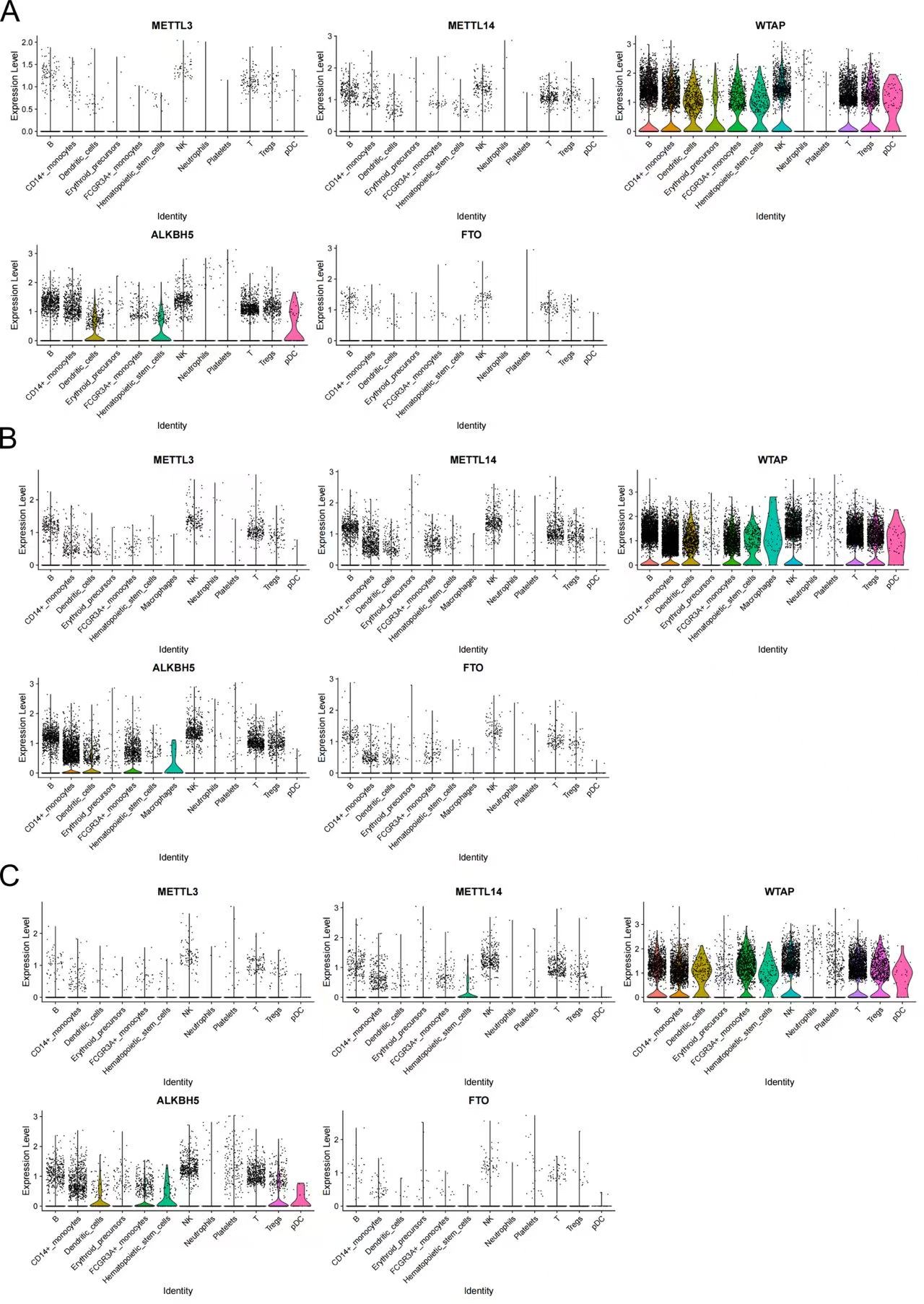

Supplement: Supplementary file 3 [file Image3.jpeg]
